# Investigating trends in interest for benign prostatic hyperplasia surgery options using Google Trends

**DOI:** 10.1038/s41391-023-00692-0

**Published:** 2023-07-08

**Authors:** James P. Daniels, Devin N. Patel, Gloria Cecilia Galvan, Nadine A. Friedrich, Sanjay Das, Arash Akhavein, Timothy Daskivich, David Josephson, Premal Desai, Cosimo De Nunzio, Stephen J. Freedland

**Affiliations:** 1 Cedars-Sinai Medical Center, Los Angeles, CA USA; 2 The Urology Center of Colorado, Denver, CO USA; 3 University of California- Los Angeles, Los Angeles, CA USA; 4 Sapienza University of Rome, Roma, Italy; 5 Durham VA Medical Center, Durham, NC USA

**Keywords:** Prostatic diseases, Medical research

## Abstract

Understanding patient interest among surgical options is challenging. We used Google Trends to analyze interest in benign prostatic hyperplasia (BPH) surgeries recommended for prostate volumes <80 cc. Google Trends was queried with five BPH surgeries. Final rank of search terms was TURP, UroLift, Rezum, Aquablation, and Greenlight. Google Trends can be an effective tool for evaluating public interest trends in BPH surgery.

Classically, transurethral resection of the prostate (TURP) has been the gold standard for benign prostatic hyperplasia (BPH) surgery. From 2013 to 2019, in the United States Medicare population, 703,919 BPH procedures were performed and TURP was the most common [1]. Currently, over ten options exist depending on surgeon expertise, patient preference, available resources, and prostate size and/or anatomy [2]. Indeed, the advent of these alternative surgeries corresponded to a drop in utilization of TURP. TURP represented 51% of BPH procedures in 2013 but only 42% in 2019. Conversely, use of less-invasive BPH procedures increased. For example, UroLift represented 1.5% of BPH procedures in 2015, but 23.7% in 2019.

Given the plethora of surgical options, understanding current preferences and growing trends is essential. One approach to measure interest is using internet search data from Google Trends. We previously analyzed Google Trends data to gauge public interest in supplements for prostate cancer prevention [3]. Merheb et al. used Google Trends data to examine interest in five BPH surgeries from July 2015 to February 2019 [4]. Search terms “Urolift” and “Rezum” both had significant increased interest over the study period compared to “TURP”, “HoLEP”, and “photoselective vaporization of the prostate” in the United States. However, as these data are nearly 3 years old, we updated this analysis to include more recent data, provide a longer historical look, and include one newer option (Aquablation), which was not included in the prior analysis.

Google Trends (http://trends.google.com) was queried for five BPH surgeries recommended by AUA guidelines for prostate volumes <80cc using search terms: “TURP”, “Rezum”, “Urolift”, “Aquablation”, and “Greenlight Laser Therapy”. “Greenlight Laser Therapy” was entered as a search topic because the surgery is referred to by multiple names. Geographical location was set to “United States”. Time range was set to “1/1/2010 – 10/31/2022”. Category section remained “All categories”. Search was limited to “Web Search”. Data are presented as relative search interest (RSI) by each month of the time range. RSI ranges 0–100 with 100 representing peak popularity, 50 representing half of peak, and 0 representing insufficient data for determination. RSI data of the five BPH procedures from January 2010 to October 2022 are shown (Fig. 1).

**Fig. 1:**
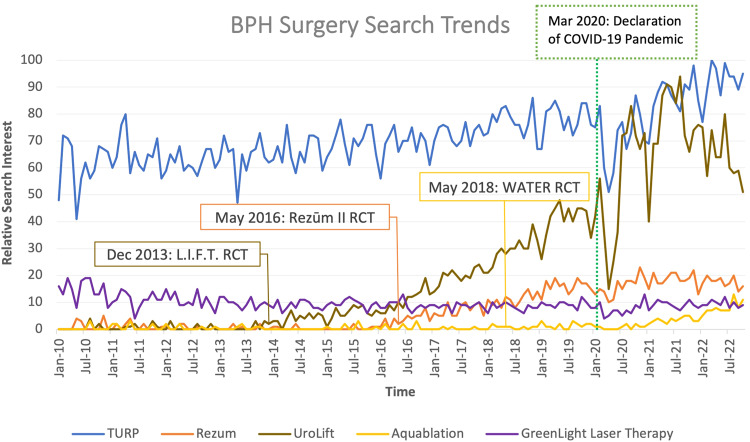
Relative search interest of five BPH surgeries from January 1, 2010 to October 31, 2022 with dates of RCT publication and start of COVID pandemic.

Results are limited to search interest in United States and may not reflect interest in Europe, Asia, or Oceania. Steady increases of searches for “Urolift” and “Rezum” began after publication of randomized control trials (RCTs) demonstrating notable lower urinary tract symptoms (LUTS) relief while preserving erectile and ejaculatory function [5, 6]. Although less distinct in comparison, increased searches for “Aquablation” began after publication of an RCT showing similar efficacy to TURP with less risk of anejaculation [7].

“Urolift” RSI trended upward following publication of L.I.F.T, the first multicenter RCT using prostatic urethral lift to treat BPH, in December 2013. Before L.I.F.T publication, from January 2010 to December 2013, average “Urolift” RSI was 0.60. After publication, from January 2014 to October 2022, average “Urolift” RSI was 33.39 with a peak of 94 in August 2021.

“Rezum” RSI trended upward following publication of the first multicenter Rezum RCT to treat BPH in May 2016. Before this RCT, from January 2010 to May 2016, average “Rezum” RSI was 0.71. After publication, from June 2016 to August 2022, “Rezum” average RSI was 13.39, with a peak of 23 in November 2020.

“Aquablation” RSI gradually trended upward following publication of the WATER RCT in May 2018. From January 2010 to May 2018, average “Aquablation” RSI was 0.45. After publication, from June 2018 to October 2022, average “Aquablation” RSI was 2.9 with a peak RSI of 13 in August 2022.

The final rank of search terms by RSI were: “TURP”, “Urolift”, “Rezum”, “Aquablation”, and “Greenlight laser therapy”. “Greenlight laser therapy” RSI had no notable trend, and the overall average RSI was 9.65. “TURP” retained the top RSI, reaching a peak of 100 in March 2022 and an overall average RSI of 71.48.

Upward RSI trends for “Urolift” and “Rezum” suggest public interest may be veering toward outpatient procedures that provide better recovery and decreased sexual dysfunction. In a similar study, from July 2015 to February 2019, “Urolift” and “Rezum” both had significantly increasing popularity [4]. Our data extend these findings and note interest in UroLift continued to climb and now rivals TURP, though interest in UroLift declined in the past year, whereas Rezum remained relatively flat over the past 2 years. Moreover, upward trends of “Urolift” RSI coincide with increasing use of UroLift seen in American Board of Urology (ABU) case logs from 2008 to 2021 [8]. A recent cross-sectional study analyzing BPH surgical option choice using ABU case logs found that amongst included surgical options, logs of UroLift increased from 1.6% in 2015 to 32.5% by 2020. This suggests our observed increased RSI may correlate with increased use, pending further confirmation.

“Aquablation” interest was relatively low but recent increased RSI may lead to similar upward trends as “Urolift” or “Rezum. “Greenlight laser therapy” interest was surpassed by more novel procedures. Further research comparing these Google Trends data and verified rates of BPH surgeries received are warranted.

Investigations into patient preference yield varied results. A 2020 systematic review found patient preferences for BPH surgery were for low-risk management options, fewer sexual side effects, and improved urge incontinence and nocturia [9]. Whereas a more recent cross-sectional survey found complication risk had the highest relative importance, intriguingly, ejaculatory dysfunction was the lowest relative importance [10]. Though patients may prefer low complication risk overall, other preferences such as efficacy vs sexual side effects can differ depending on variables like age, LUTS severity, and surgical history. Google Trends cannot be used to measure receipt of surgical treatments, but it is a novel tool for evaluating public interest in BPH surgery as new options and long-term RCT results are made available.
